# MBD3 promotes hepatocellular carcinoma progression and metastasis through negative regulation of tumour suppressor TFPI2

**DOI:** 10.1038/s41416-022-01831-5

**Published:** 2022-04-30

**Authors:** Weiwei Yan, Qiuying Han, Lin Gong, Xiaoyan Zhan, Wanjin Li, Zenglin Guo, Jiangman Zhao, Tingting Li, Zhaofang Bai, Jin Wu, Yan Huang, Luye Lv, Haixin Zhao, Hong Cai, Shaoyi Huang, Xinwei Diao, Yuan Chen, Weili Gong, Qing Xia, Jianghong Man, Liang Chen, Guanghai Dai, Tao Zhou

**Affiliations:** 1 State Key Laboratory of Proteomics, Institute of Basic Medical Sciences, National Center of Biomedical Analysis, 100850 Beijing, China; 2 Department of Radiation Oncology, 5th Medical Center of Chinese PLA General Hospital, 100853 Beijing, China; 3 Nanhu Laboratory, 314002 Jiaxing, Zhejiang Province China; 4 Department of Hepatobiliary Surgery, PLA navy No. 971 Hospital, 266071 Qingdao, Shandong Province China; 5 Department of Liver Disease, 5th Medical Center of Chinese PLA General Hospital, 100039 Beijing, China; 6 Department of Oncology, 5th Medical Center of Chinese PLA General Hospital, 100853 Beijing, China

**Keywords:** Oncogenes, Cell invasion

## Abstract

**Background:**

The mechanism of recurrence and metastasis of hepatocellular carcinoma (HCC) is complex and challenging. Methyl-CpG binding domain protein 3 (MBD3) is a key epigenetic regulator involved in the progression and metastasis of several cancers, but its role in HCC remains unknown.

**Methods:**

MBD3 expression in HCC was detected by immunohistochemistry and its association with clinicopathological features and patient’s survival was analysed. The effects of MBD3 on hepatoma cells growth and metastasis were investigated, and the mechanism was explored.

**Results:**

MBD3 is significantly highly expressed in HCC, associated with the advanced tumour stage and poor prognosis in HCC patients. MBD3 promotes the growth, angiogenesis and metastasis of HCC cells by inhibiting the tumour suppressor tissue factor pathway inhibitor 2 (TFPI2). Mechanistically, MBD3 can inhibit the *TFPI2* transcription via the Nucleosome Remodeling and Deacetylase (NuRD) complex-mediated deacetylation, thus reactivating the activity of matrix metalloproteinases (MMPs) and PI3K/AKT signaling pathway, leading to the progression and metastasis of HCC

**Conclusions:**

Our results unravel the novel regulatory function of MBD3 in the progression and metastasis of HCC and identify MBD3 as an independent unfavourable prognostic factor for HCC patients, suggesting its potential as a promising therapeutic target as well.

## Background

Hepatocellular carcinoma (HCC) is the sixth most common malignant tumour, and the fourth most common cause of cancer-related death worldwide [1]. At present, the comprehensive treatment based on surgery is still the most effective treatment for HCC. However, due to its hidden features, most patients are diagnosed at advanced stages and do not qualify for radical surgery. More disappointedly, since most patients with HCC have underlying liver diseases, such as chronic hepatitis and cirrhosis, the recurrence rate within 5 years post-surgery is as high as 70%, and the total survival rate is only 25–40% [2, 3]. Therefore, elucidating the molecular mechanisms of HCC progression could identify novel markers to facilitate early diagnosis as well as targets for future clinical therapies.

Epigenetic regulation plays an increasingly important role in the occurrence and development of HCC [4]. Methyl-CpG binding domain (MBD) protein family is a major member of epigenetics, which can recognise and bind methylated DNA, and stabilise transcription inhibition by histone deacetylases (HDAC), thus ensuring gene silencing [5]. MBD3 is a key member of the MBD protein family, characterised by a methylcytosine binding domain and gene-repressing activity [6]. It can directly bind to 5-hydroxymethylcytosine-containing DNA or bind methylated DNA indirectly through other bound proteins; consequently, regulating genes with these modifications on the promoter [7]. The primary function of MBD3 is to exist as the core subunit in the Nucleosome Remodeling and Deacetylase (NuRD) complex and exert transcriptional regulatory function through other subunits [8]. MBD3 plays a key role in the pluripotency, differentiation and development of embryonic stem cells (ESC) through NuRD complex; MBD3 knockout mice exhibit embryonic lethality [9]. MBD3 can also participate in the occurrence and development of tumours. Its specific role in tumours depends on the tumour type, microenvironment and other factors [10]. Previous studies have reported that MBD3 has the highest cancer mutation rate in pancreatic cancer, and some research has shown that MBD3 has an anti-tumour effect in pancreatic cancer; specifically, it can inhibit the EMT of pancreatic cancer cells through TGF-β/smad signaling pathway [11]. Moreover, it has been reported that upregulated MBD3 facilitates breast cancer progression through transcription activation of HIF2a [12]. However, the role of MBD3 in HCC and its association with the progression of the disease remain unclear.

In this study, we evaluated MBD3 expression levels in HCC patients’ samples and analysed their correlations with the clinical prognosis. We then explored MBD3’s role in HCC cells proliferation and invasion in vivo and in vitro, and further identified the downstream target genes regulated by MBD3 and elucidated its underlying mechanism.

## Materials and methods

### HCC samples and immunohistochemistry (IHC)

We collected 227 cases of HCC tissue and adjacent liver tissues after hepatectomy from the Fifth Medical Center of the Chinese PLA General Hospital, Beijing. This study was conducted ethically in accordance with the World Medical Association Declaration of Helsinki and was approved by the Ethics Committee of the Chinese PLA General Hospital. Written informed consent was obtained from all patients participated. All cases were confirmed by pathology and followed up for >5 years with complete clinical data. Clinical human samples were paraffin-embedded, fabricated into tissue microarrays, and cut into 5 μm paraffin slices. After xylene and gradient ethanol soaking, antigen repair, elimination of endogenous peroxidase, and blocking with 5% goat serum, the slices were incubated with the primary antibody at 4 °C overnight. Then, the slices were incubated with the second antibody for 30 min at room temperature, stained with DAB, and nucleated with hematoxylin. After dehydration in gradient ethanol and sealing with neutral gum, two pathologists independently read and scored the MBD3 expression using “H-score” method: H-score = The proportion score (PS) × The intensity score (IS) [13]. PS was calculated as 0 (<5%), 1 (6–25%), 2 (26–50%), 3 (51–75%) or 4 (>76%). IS was calculated as 0 (no staining), 1 (weak staining), 2 (medium staining) or 3 (strong staining). The cut-off value for high or low-MBD3 expression was a median of 6. All the primary antibodies used in IHC were listed in the Supplementary information (Supplementary Table S1).

### Cell culture and plasmids

The human embryonic kidney cells HEK293, hepatocytes L02 and liver tumour-derived cell lines (Hep3B, SK-Hep1 and HepG2) were purchased from the American Type Culture Collection. MHCC97H and Huh7 were obtained from the Cell Bank of Type Culture Collection of Chinese Academy Sciences (Kunming, China). All the cell lines were cultured in RPMI1640 or DMEM with 10% fetal bovine serum in the cell incubator containing 5% CO_2_ at 37 °C. All the primers used in the plasmids were listed in the Supplementary information (Supplementary Table S2).

### Cell proliferation—MTS

Cells were cultured in a 96-well plate with 1000 cells/200 μL complete medium in each well, with triplicate wells for each cell. Twenty microlitres of MTS solution was added to each well at the same time every day. After incubation for 2–4 h, 490 nm absorption of each well was measured by an enzyme labelling instrument, and the growth curve was plotted.

### Cell invasion—transwell

Matrigel glue was purchased from MC company and Transwell orifice test was purchased from Corning. Firstly, the Matrigel was spread on the inner membrane of the chamber, and then the cells were cultured in each chamber with 4 × 10^4^ cells in 100 μL serum-free medium and 500 μL complete medium in the lower holes. After being cultured in 5% CO_2_ at 37 °C for 24 h, the inner cells were removed with cotton swabs; then, the outer cells penetrating the chamber were immersed in 75% ethanol for 15 min, followed by 0.4% crystal violet solution for 15 min, and finally in distilled water 2–3 min for three times. After drying, the bottom cells were scanned, photographed and counted.

### Animal experiment

SPF grade BALB/c male nude mice aged 4–6 weeks were used for subcutaneous tumorigenesis and pulmonary metastasis. For the subcutaneous tumorigenesis, tumour cells were injected subcutaneously into the right hip of nude mice with 4 × 10^6^ cells in 100 μL serum-free DMEM for each mouse, seven mice/group. The maximum diameter (L) and minimum diameter (W) of the subcutaneous tumours were measured every 3 days. The volume of the tumours was calculated according to the formula V = L × W^2^/2, and the growth curve was recorded. After 4 weeks, the nude mice were sacrificed and the subcutaneous tumours were removed, photographed and weighed.

For the pulmonary metastasis, tumour cells were injected into nude mice via the tail vein with 2 × 10^6^ cells in 100 μL PBS solution for each mouse, three mice/group. After 2 h, luciferase substrates (concentration 15 mg/mL) were injected into the abdomen of each mouse at the dose of 10 μL /g body weight. The nude mice were then anaesthetised by isoflurane inhalation and their lung conditions observed on a IVIS Living Image instrument. The formation of pulmonary metastases was detected as above in vivo from weeks 1–4 and the luciferase luminescence curve was drawn.

The animal experiments were approved by the Institutional Animal Care and Use Committee of the Institute of National Center of Biomedical Analysis and performed in accordance with this committee’s guidelines.

### Western blotting

Proteins were prepared from cells by lysis in M2 buffer (20 mM Tris-HCl (pH 7.5), 250 mM NaCl, 0.5% NP-40, 3 mM EDTA, 3 mM EGTA) supplemented within PMSF, phosphatase inhibitor cocktails and DTT. They were diluted in 2× Loading buffer and boiled in the metal bath at 105 °C for 10 min, then to be analysed by western blot. All the primary antibodies used in this study are listed in the Supplementary information (Supplementary Table S1).

### Co-immunoprecipitation (Co-IP)

Huh7 cells were lysed in M2 Buffer (20 mM Tris-HCl (pH 7.5); 250 mM NaCl; 3 mM EDTA; 3 mM EGTA and 0.5% NP-40) with PMSF, phosphatase inhibitor cocktails and DTT; rotating at 4 °C for 30 min. After centrifugation, 40 μL of the supernatant was reserved as Input, and then incubated with the primary antibody or control IgG, respectively, rotating overnight at 4 °C. The next day, protein A/G beads were added, and the mixture was incubated rotationally at 4 °C for 2 h. The beads were then washed with M2 buffer four times. The proteins bound to the beads were analysed by western blot.

### Chromatin immunoprecipitation (ChIP)

The ChIP assay was performed using the SimpleChIP Enzymatic Chromatin IP kit (#9002, Cell Signaling Technology). Briefly, Cells were fixed with 1% formaldehyde and lysed. After protein-DNA decrosslinking, ChIP assay was performed using the specific antibodies. The crosslinks were then reversed, and DNA was purified. The enrichment of specific DNA sequences was analysed by qPCR. Histone H3 antibody was used as the positive control and normal rabbit IgG antibody as the negative control. All the antibodies and primers used in the ChIP were listed in the Supplementary information (Supplementary Tables S1, S3)

### Statistical analysis

SPSS 21.0 software and GraphPad Prism 5.0 were used for statistical analysis. All experiments were conducted at least in triplicate. Mann–Whitney *U* tests were used to analyse the differences in MBD3 expression between HCC tissues and normal liver tissues. Pearson Chi-square test or Fisher’s Exact test were used to analyse the relationship between the clinicopathological data and MBD3 expression in the 227 HCC patients. The Kaplan–Meier curve method and Log-Rank test were used to plot the disease-free survival (DFS) and overall survival (OS) of HCC patients. Unpaired Student’s *t-*tests or Welch’s *t*-tests were used to analyse the statistical significance between the two groups. The data were shown as mean ± SD or mean ± SEM and *P* < 0.05 was considered statistically significant (*P < 0.05; **P < 0.01; ***P < 0.001).

For further details regarding the materials and methods used, please refer to the supplementary information (Supplementary Table S1-3).

## Results

### MBD3 is highly expressed in HCC and indicates a poor prognosis

**Fig. 1:**
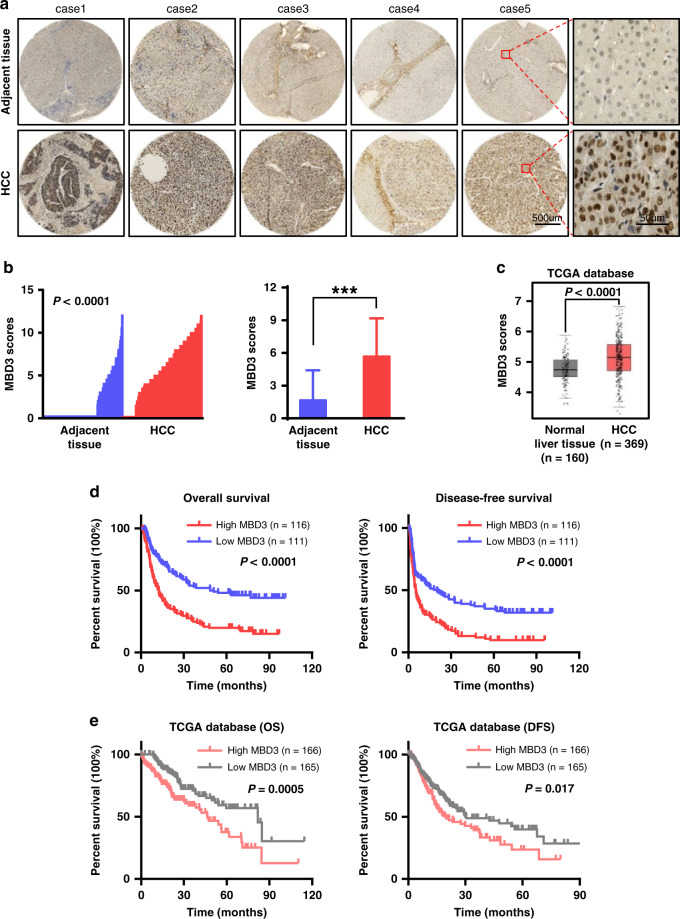
MBD3 is highly expressed in HCC and indicates poor prognosis. **a** Representative IHC staining of MBD3 in HCC and adjacent tissues, with normal IgG as a negative control. Scale bars: 500 μm and 50 μm. **b** Left panel: The MBD3 scores of each liver cancer and adjacent liver tissue. Right panel: The MBD3 scores of HCC and adjacent liver tissues (*n* = 227), expressed as mean ± SD. **c** Analysis of MBD3 mRNA levels in normal liver tissue (*n* = 160) and HCC (*n* = 369) from the TCGA database. **d**, **e** Kaplan–Meier curves of overall and diseases-free survival of 227 HCC samples we collected (**d**) and 331 HCC samples from the TCGA database (**e**), stratified by MBD3 expression. **b**, **c** Mann–Whitney *U* test; **d**, **e** log-rank test. ***P < 0.001.

We collected 227 cases of primary HCC with radical resection and performed MBD3-specific antibody IHC analysis. The results showed that MBD3 protein was primarily located in the nucleus of hepatoma cells and normal hepatocytes, and its expression levels were significantly upregulated in hepatoma tissues compared with the adjacent normal liver tissues (*P* < 0.0001, Fig. 1a, b, left panel). IHC score staining of the 227 cases showed that the average expression intensity of MBD3 in the adjacent normal liver tissue was 1.655 ± 2.748, while the average in the hepatoma tissues was 5.667 ± 3.504 (Fig. 1b, right panel). Upregulation of MBD3 in HCC was also confirmed in the TCGA dataset (Fig. 1c).

We then further analysed the relationship between clinicopathologic features and MBD3 expression levels in the HCC cases. MBD3 expression was significantly correlated with preoperative AFP level (*P* < 0.001), vascular invasion (*P* = 0.013), tumour capsule (*P* = 0.013), Edmondson grade (*P* = 0.005) and TNM-stage (*P* = 0.001). The MBD3 expression was not significantly correlated with gender, age, drinking history, hepatitis B, liver cirrhosis, lymph node metastasis or tumour size and number (Supplementary Table S4). Most important, the univariate and multivariate analysis of factors associated with survival in 227 HCC patients by Cox regression model demonstrated that MBD3 expression was an independent unfavourable prognostic factor for OS (*P* < 0.001) and DFS (*P* = 0.002). (Supplementary Table S5, 6).

Furthermore, Kaplan–Meier survival analysis was used to compare OS / DFS of patients with the high- and low-MBD3 expression (cut-off = the median value (6) of IHC score). The median OS of the high-MBD3 group was significantly decreased than the low-MBD3 group (12.04 vs 51.03 months, *P* < 0.0001); the median DFS of the high-MBD3 group was also obviously decreased compared with the low-MBD3 group (5.13 vs 20.07 months, *P* < 0.0001). These results indicate that MBD3 expression level is closely correlated to the poor prognosis of HCC patients (Fig. 1d). Consistently, high-MBD3 expression was associated with worse outcomes in the TCGA dataset (Fig. 1e). Taken together, these clinical data suggest that hepatomas with high-MBD3 expression were more prone to relapse and metastasis.

### MBD3 promotes HCC cell growth and metastasis in vitro and in vivo

**Fig. 2:**
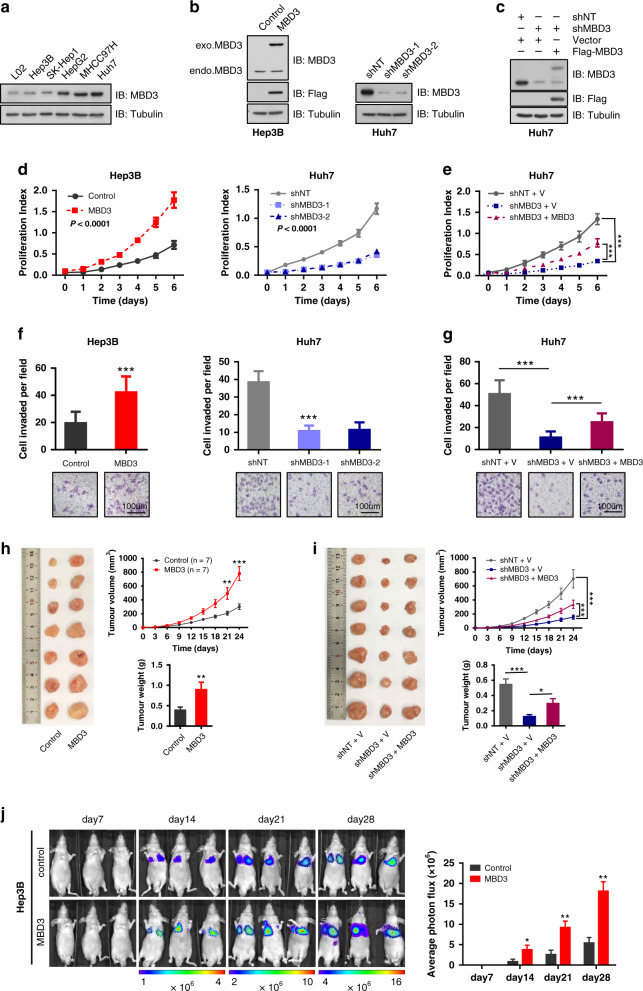
MBD3 promotes HCC cell growth and metastasis in vitro and in vivo. **a** Western blotting of MBD3 in normal hepatocyte L02 and other five HCC cell lines. **b** Flag-MBD3 was stably transfected in Hep3B cells and Huh7 cells were stably infected with lentivirus expressing control or MBD3 shRNA (−1 and −2), protein expression levels were analysed by western blotting. **c** Resistant Flag-MBD3 was stably transfected into Huh7-shMBD3 cells and the indicated protein expression levels were detected by western blotting. **d**, **e** The effect of MBD3 on the growth of HCC cells was detected by MTS assay. **f**, **g** The effect of MBD3 on the invasion of HCC cells was detected by transwell assay. Scale bar: 100 μm. **h**, **i** Representative image of the subcutaneous tumorigenesis in nude mice, tumour weight and tumour growth curve of each group (*n* = 7/group) were analysed. **j** Hep3B-control or Hep3B-MBD3 cells were injected into the tail vein of nude mice (*n* = 3/group) to detect lung metastasis, and the fluorescence intensity from week 2–4 was measured. **d**, **e**, **j** Mean ± SD, two-way ANOVA; **f**–**i** (upper panel) Mean ± SD, or **h**, **i** (lower panel) Mean ± SEM, unpaired Student’s *t*-test or Welch’s *t*-test. *P < 0.05, **P < 0.01, ***P < 0.001.

In order to investigate the role of MBD3 in the development of HCC, we first estimated the MBD3 expression level in a normal hepatocyte cell line (L02) and five HCC cell lines (Hep3B, SK-Hep1, HepG2, MHCC97H and Huh7) (Fig. 2a). Then, we stably overexpressed MBD3 in Hep3B cells and knocked down MBD3 in Huh7 cells (Fig. 2b). To better understand the role of MBD3 in HCC cells, we also stably reintroduced shRNA-resistant MBD3 into the Huh7-shMBD3 cells (Fig. 2c). Next, we completed a series of cell proliferation and invasion experiments in vitro and in vivo using the above-constructed hepatoma cell lines. Overexpression of MBD3 in Hep3B cells dramatically increased the cell proliferation (*P* < 0.0001) and plate colony formation (*P* < 0.0001). Conversely, knockdown by two different shRNAs targeting MBD3 (shMBD3-1 and −2) in Huh7 cells significantly decreased cell growth and clonogenicity (Figs. 2d and S1a). However, the proliferation inhibition caused by MBD3 depletion was largely rescued by the reintroduction of shRNA-resistant MBD3 into MBD3-knockdown Huh7 cells (Fig. 2e). These results indicate that MBD3 promotes the proliferation of hepatoma cells. Moreover, soft agar colony-forming results revealed that the ability of Hep3B cells to form colonies in soft agar was largely enhanced with MBD3 expression and significantly impaired after MBD3 depletion, suggesting that MBD3 plays a role in tumour transformation (Fig. S1b).

Based on our findings that MBD3 might play an oncogenic role in the development of HCC, we postulated that it may also be associated with alterations in migration and invasion: two biological actions essential for tumour metastasis. We first conducted the transwell assays and found that the migratory and invasive capacity of Hep3B cells transfected with MBD3 was obviously enhanced compared to the control cells, while that of Huh7 cells with MBD3 interference was significantly perturbed (Fig. S1c and Fig. 2f). But the invasiveness inhibition in the MBD3-knockdown Huh7 cells can be rescued accompanied by reintroducing shRNA-resistant MBD3 (Fig. 2g). The wound-healing assay exhibited analogous results (Fig. S1d). These results indicate that MBD3 promotes the migration and invasion of hepatoma cells.

To further evaluate the impact of MBD3 on the growth and metastasis of hepatoma cells in vivo, firstly we carried out subcutaneous tumorigenesis experiments in BALB/c nude mice. The results showed that MBD3 overexpression in Hep3B cells led to a significant increase in tumour weight and volume (Fig. 2h). The same results showed in Huh7 cells, when MBD3 was knocked down, both the volume and weight of subcutaneous tumours were significantly reduced and were partially recovered when MBD3 was re-expressed (Fig. 2i). Next, we established a metastasis model using Hep3B-control and -MBD3 cells with a luciferase reporter. The results showed that the lung metastasis appeared from the second week in BALB/c mice. Notably, the intensity and area of lung signals representing lung metastasis in MBD3 overexpression group were higher than in the control group from weeks 2–4 and the difference between the two groups became greater as time extended (Fig. 2j). Collectively, these in vitro and in vivo findings support the supposition that MBD3 might play an oncogenic role in the development of HCC.

### Tumour suppressor TFPI2 is the downstream target gene regulated by MBD3

**Fig. 3:**
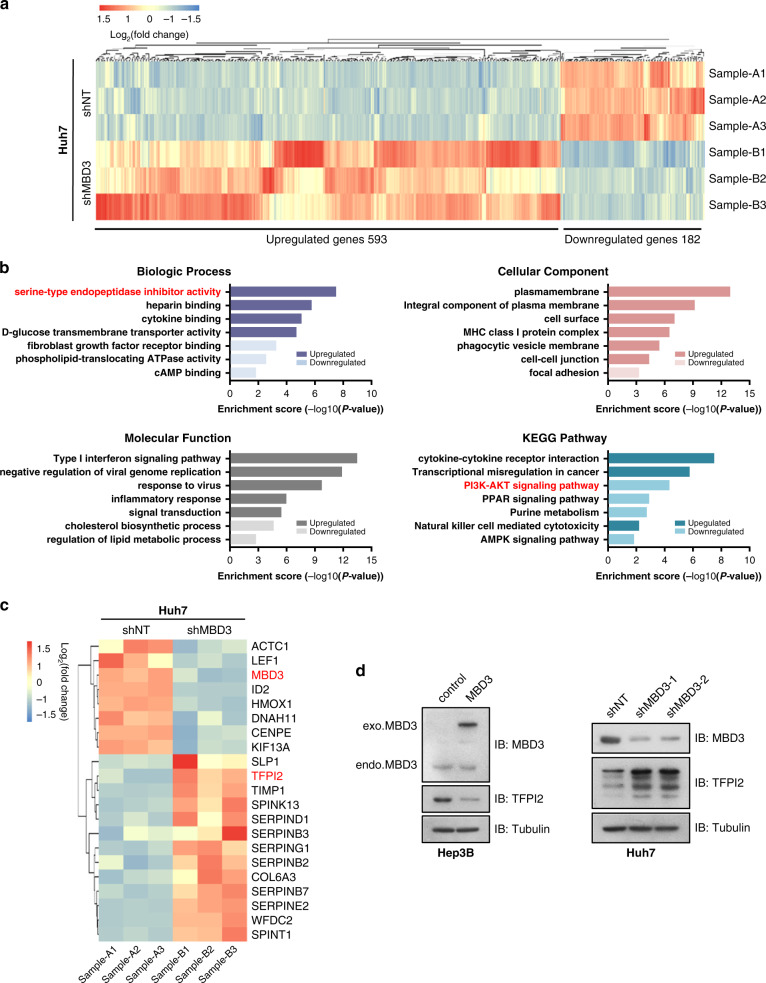
Tumour suppressor TFPI2 is the downstream target gene regulated by MBD3. **a** Huh7 cells stably transfected with shMBD3 and control were analysed by the whole-transcriptome sequencing (RNA-seq). All the altered genes after MBD3 knockdown are shown (1.5-fold, *P* < 0.05). **b** GO and KEGG enrichment analysis of the above differential genes were performed by DAVID database, and top 7 term regulated by MBD3 in biologic process, cellular component, molecular function as well as KEGG pathway were shown. **c** The heatmap of differential genes in the serine endopeptidase inhibitor family caused by MBD3 inhibition in Huh7 cells. **d** Western blotting of MBD3 and TFPI2 in the indicated stable cell lines.

To identify the downstream target genes participating in tumour regulation in MBD3-mediated HCC, we compared the whole gene transcriptional profiles of Huh7-shNT cells and -shMBD3 cells by RNA-seq. A total of 775 genes differentially regulated by MBD3 knockdown (1.5-fold, *P* < 0.05) were identified, among which 593 were upregulated and 182 were downregulated (Fig. 3a). GO enrichment analysis revealed that several biologic processes, cellular components and molecular functions were affected by MBD3 knockdown; notably, there is an obvious increase of serine-type endopeptidase inhibitor activity after MBD3 knockdown, while the PI3K-AKT signaling pathway is significantly downregulated analysed by the KEGG enrichment (Fig. 3b). Serine-type endopeptidase inhibitors can stabilise extracellular matrices by directly inhibiting the activity of metallopeptidase and reducing cell focal adhesion, subsequently inhibiting the metastasis of tumour cells. We speculated that MBD3 may promote HCC growth and metastasis by regulating serine-type endopeptidase inhibitor activity. We further screened the different genes in the serine-type endopeptidase inhibitors and found that Tissue factor pathway inhibitor 2 (TFPI2) increased significantly after MBD3 interference (Fig. 3c).

TFPI2 is a Kunitz-type serine proteinase inhibitor that has been identified as a tumour suppressor. It can reduce extracellular matrix degradation by inhibiting metalloproteinase, stabilising plasma membranes as well as basement membranes and inhibiting the metastasis of tumour cells [14, 15]. We confirmed the association between MBD3 and TFPI2 by western blotting. The results showed that MBD3 overexpression in Hep3B cells decreased TFPI2 protein expression, whereas knockdown of MBD3 in Huh7 cells led to TFPI2 upregulation (Fig. 3d). These results suggest that MBD3 may promote the growth and metastasis of liver cancer by inhibiting the downstream gene TFPI2.

### MBD3 promotes the angiogenesis of HCC tissues by inhibiting TFPI2

**Fig. 4:**
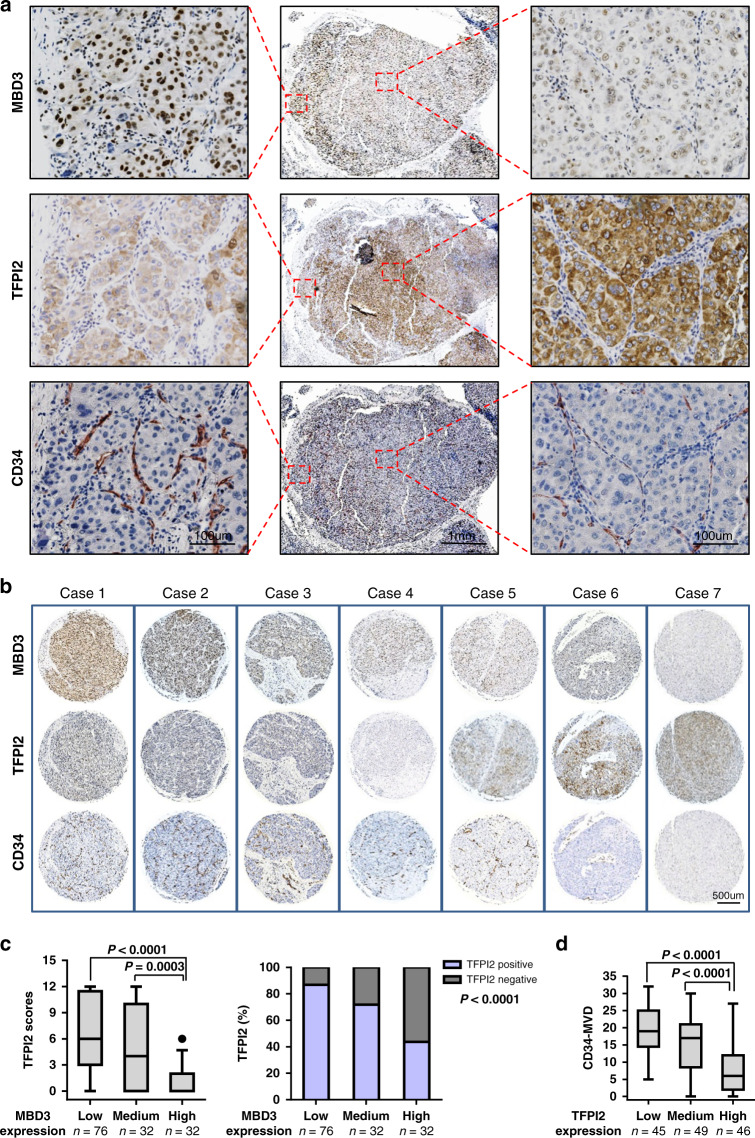
MBD3 promotes the angiogenesis of HCC tissues by inhibiting TFPI2. **a** Representative IHC staining of MBD3, TFPI2 and CD34 in different parts of serial sections of HCC. Scale bars: 100 μm and 1 mm. **b** Representative IHC staining of MBD3, TFPI2 and CD34 in serial sections of 140 HCC cases. Scale bar: 500 μm. **c** Boxplot showing TFPI2 expression (left panel) and the percentage of TFPI2–positive tumours (right panel) in 140 HCC samples. The samples were divided into three groups according to MBD3 expression scores in the tumours, representing low (scores 0–4), medium (scores 5–8) and high (scores 9–12) expression of MBD3. **d** The relationship of CD34-MVD and TFPI2 in three groups of samples divided based on TFPI2 expression scores as that of MBD3 in **c**. Data were analysed by one-way ANOVA test (**c** (left panel) and **d**) and Pearson’s χ^2^ test (**c** (right penal)).

In order to investigate the clinical significance of the above findings, we carried out immunostaining of MBD3 and TFPI2 in consecutive clinical specimens of human HCC samples. Since it has been reported that TFPI2 may inhibit tumour metastasis by inhibiting the angiogenesis of tumour cells [16], we measured CD34 levels as well. The results revealed that TFPI2 was expressed in the cytoplasm. Notably, staining of consecutive clinical specimens showed that the MBD3 expression was negatively correlated with the TFPI2 expression, which in turn was negatively correlated with CD34 expression (Fig. 4a). Analogous results were found in other consecutive paraffin sections of 140 HCC clinical samples (Fig. 4b).

Moreover, we divided these samples into three groups based on MBD3 amounts, as defined by expression scores, and evaluated the TFPI2 expression among these three groups. The results showed that TFPI2 expression in the high-MBD3-expression group was significantly decreased compared with those in the low- or medium-MBD3-expression group (Fig. 4c), further confirming that MBD3 expression has an inverse correlation with TFPI2 in HCC.

We also assessed the microvessel density (MVD), which is considered to be an indicator of the neoangiogenic process, by staining with a CD34 monoclonal antibody [17]. To better understand the correlation between TFPI2 expression and tumour angiogenesis, we divided these samples into three groups based on TFPI2 amount, as defined by expression scores, and studied the CD34-MVD among these three groups. The results revealed that the CD34-MVD value in the high-TFPI2-expression group was significantly decreased compared with those in the low- or medium-TFPI2-expression group (Fig. 4d). CD34-MVD was found to be inversely correlated with TFPI2 expression, indicating that TFPI2 inhibits angiogenesis.

Taken together, these suggest that MBD3 inhibits the expression of the tumour suppressor TFPI2, thereby undoing its inhibition of tumour angiogenesis, eventually promoting the progression and metastasis of HCC.

### MBD3 promotes the proliferation and invasion of hepatoma cells by inhibiting TFPI2

**Fig. 5:**
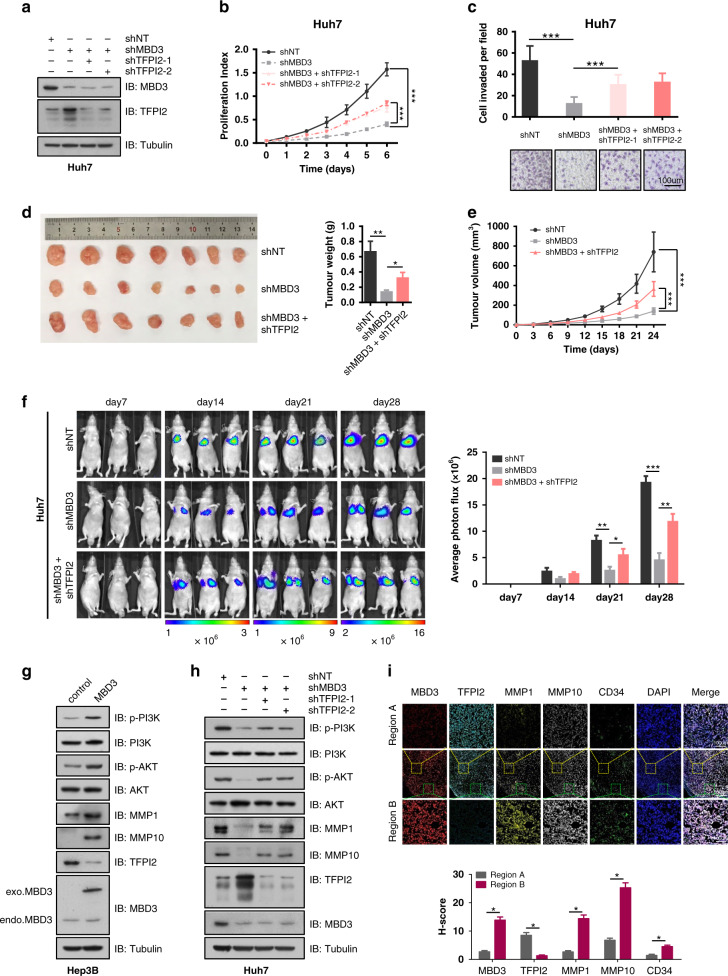
MBD3 promotes the proliferation and invasion of hepatoma cells by inhibiting TFPI2. **a** Huh7 cells were stably transfected with shNT, shMBD3 or shMBD3 + shTFPI2, and the indicated protein expression levels were detected by western blotting. **b**, **c** The proliferation and invasion ability of the cells were tested by MTS assay (**b**) and transwell assay. Scale bar: 100 μm (**c**). **d**, **e** Representative image of the subcutaneous tumorigenesis in nude mice. Tumour weight (**d**) and tumour growth curve (**e**) of the indicated groups (*n* = 7/group) were analysed. **f** The indicated cells were injected into the tail vein of nude mice to detect lung metastasis. The fluorescence intensity of each group (*n* = 3/group) was measured from week 2–4. **g**, **h** Western blot analysis of the expression levels of p-PI3K, p-AKT, MMPs and TFPI2 in MBD3 overexpressing/knockdown and TFPI2/MBD3 duo-knockdown cells. **i** Representative fluorescent staining of MBD3, TFPI2, MMP1, MMP10 and CD34 in different parts of the same subcutaneous tumour tissue of nude mice. **b**, **e** Mean ± SD, two-way ANOVA; **c**, **f** Mean ± SD, **d**, **i** mean ± SEM, unpaired Student’s *t*-test or Welch’s *t*-test. *P < 0.05, **P < 0.01, ***P < 0.001.

According to the clues provided by the above RNA-seq analysis and clinical research, we then investigated the possibility that TFPI2 mediated the regulation of MBD3. Consistent with data in Fig. 2, the proliferation rate and invasion capability of Huh7 cells were suppressed when MBD3 was knocked down. However, when MBD3-knockdown cells were transfected with TFPI2 shRNA (Figs. 5a and S2a), both proliferation rate and cell invasion ability significantly rebounded (Figs. 5b, c and S2b, c).

In vivo experiments yielded similar results. Subcutaneous tumorigenesis experiments in BALB/c nude mice revealed that MBD3 knockdown in Huh7 cells significantly reduced tumour growth, which was partially rescued by TFPI2 co-suppression (Figs. 5d, e and S2d, e). In vivo metastasis assays in mice using Huh7 cells with a luciferase reporter showed that the presence of Huh7 MBD3-stably-knockdown cells in the lung was significantly reduced compared to the control cells in weeks 2 to 4. However, when TFPI2 was co-knocked down, the inhibitory effect was significantly reversed (Fig. 5f).

Many TFPI2 downstream signaling pathways are affected upon MBD3 depletion, such as matrix metalloproteinases (MMPs), PI3K and AKT, which are primary factors in tumour growth and metastasis. Our results indicated that when MBD3 is highly expressed in HCC, the tumour suppressor TFPI2 would be inhibited, thus the activity of MMPs, such as MMP1, MMP10 and the PI3K/AKT signaling pathway will be reactivated, which will promote the proliferation and metastasis of tumour cells and lead to the deterioration of HCC (Figs. 5g, h and S3). This result was further verified in the subcutaneous tumour tissue of nude mice. We found that in the regions with high expression of MBD3, TFPI2 expression was decreased, while that of MMP1, MMP10 and CD34 was increased. Conversely, in the regions with low expression of MBD3, TFPI2 expression was increased, and the expression of MMP1, MMP10 and CD34 was decreased (Fig. 5i).

All these results indicate that MBD3 could promote the growth and metastasis of HCC by inhibiting tumour suppressor TFPI2.

### TFPI2 promoter can be deacetylated by the MBD3/NuRD complex

**Fig. 6:**
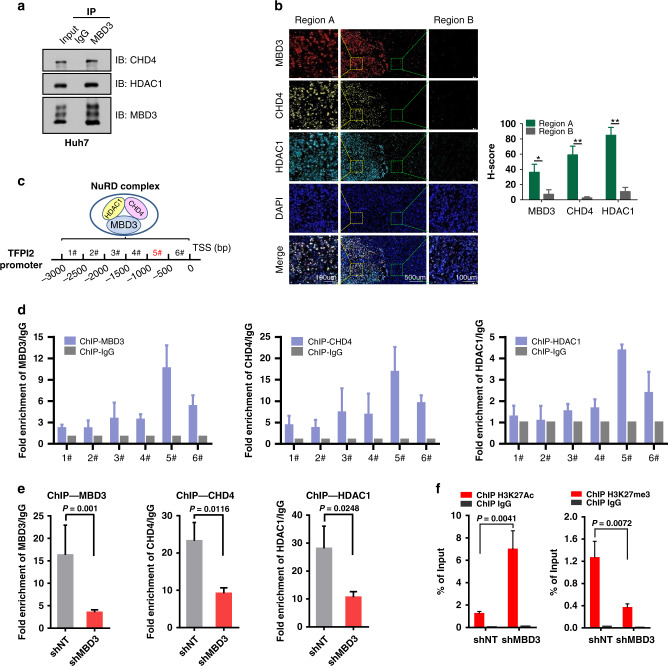
TFPI2 promoter can be deacetylated by the MBD3/NuRD complex. **a** The endogenous association of CHD4 and HDAC1 with MBD3 is detected in Huh7 cells. Immunoprecipitation (IP) was performed using anti-MBD3 antibody and the immunoprecipitates (IPs) were probed with indicated antibodies. **b** Representative fluorescence staining of MBD3, CHD4 and HDAC1 in different parts of the same human HCC tissue. **c** ChIP assay designed to detect the binding region of MBD3/NuRD complex on TFPI2 promoter. Schematic showing the amplification regions of the ChIP primers (1# to 6#) on TFPI2 promoter. **d** ChIP assay were performed with antibodies of MBD3, CHD4 and HDAC1 separately using the ChIP primers (1# to 6#), and the IPs were analysed by qPCR. **e**, **f** ChIP assays of the indicated proteins binding on TFPI2 promoter in control and MBD3-knockdown Huh7 cells. The IPs were analysed by qPCR (primer #5), assayed by unpaired Student’s *t*-test or Welch’s *t-*test.

Next, we investigated the molecular mechanisms of how MBD3 negatively regulates TFPI2 in HCC cells. Since MBD3 is the key scaffold protein of the NuRD complex and represses downstream gene transcription by recruiting Histone Deacetylases 1 (HDAC1), the major subunit of the complex, we speculated that MBD3 recruited the NuRD complex to the *TFPI2* promoter. To test this, we first verified the association of MBD3 with the other major components of the NuRD complex in HCC cells. Endogenous immunoprecipitation (IP) in HCC cells revealed that the major components of the NuRD complex, chromodomain helicase DNA binding protein 4 (CHD4) and HDAC1, were detected in the immunoprecipitates, confirming their interaction (Figs. 6a and S4a, b). Importantly, we also found the co-localisation of MBD3 with CHD4 and HDAC1 in human hepatocellular carcinoma samples (Fig. 6b). Then, we analysed the promotor of *TFPI2* in the UCSC Genome Browser Database (http://genome.ucsc.edu) and designed six primers per 500 bps (Fig. 6c).

After that, ChIP assays were performed with the indicated antibodies to detect the protein binding region on the *TFPI2* promoter. The results showed a strong enrichment of MBD3 binding around the *TFPI2* transcription start site (TSS, 5# region), and the luciferase activity of the TFPI2-luc was significantly increased in MBD3-knockdown cells compared with that of the control cells, indicating that MBD3 functions as an upstream transcriptional regulator of TFPI2 in HCC. Critically, the other subunits of the NuRD complex, CHD4 and HDAC1, were also enriched in the same region as MBD3 (Figs. 6d and S4c), and these enrichments were significantly reduced after MBD3 knockdown (Fig. 6e). We also assessed histone acetylation and methylation by detecting the enrichment of H3K27ac and H3K27me3, respectively, at the 5# region of *TFPI2* promoter. Notably, the enrichment of H3K27ac at the *TFPI2* promoter increased in MBD3-knockdown cells compared with the control cells, and the binding of H3K27me3 decreased accordingly (Figs. 6f and S5).

These data suggest that MBD3 binds to *TFPI2* promoter together with other components of the NuRD complex, resulting in *TFPI2* gene transcriptional repression via NuRD-mediated deacetylation.

**Fig. 7:**
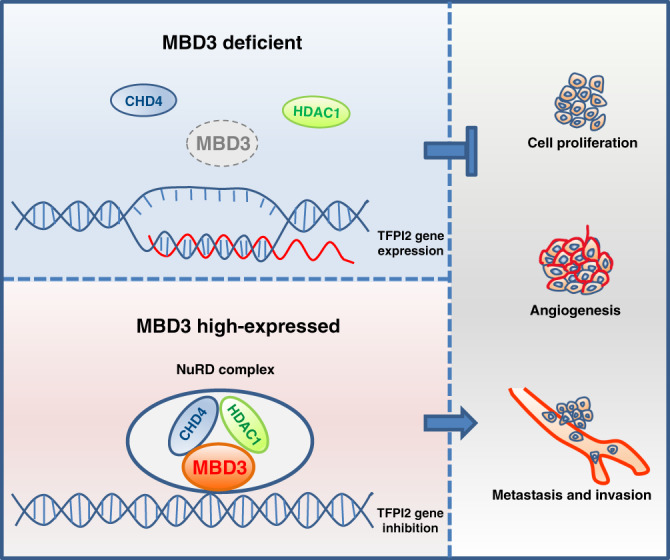
Schematic representation of MBD3 promoting HCC progression and metastasis by inhibiting TFPI2. Up panel: When MBD3 is low expressed in HCC, tumour suppressor TFPI2 can be normally expressed and inhibit the proliferation, angiogenesis and metastasis of HCC. Down panel: When MBD3 is high expressed in HCC, it can recruit CHD4 and HDAC1 to form NuRD complex and bind to the promoter of TFPI2, inducing TFPI2 gene inhibition, and then indirectly release the proliferation, angiogenesis and metastasis of HCC.

Taken together, these data show that when MBD3 is highly expressed in HCC, it can recruit CHD4 and HDAC1 to form NuRD complex and bind to the promoter of tumour suppressor *TFPI2*, and then inhibit gene transcription by histone deacetylation, so as to promote the proliferation, angiogenesis and metastasis of HCC (Fig. 7).

## Discussion

High recurrence and metastasis have always been important factors affecting the prognosis of HCC patients. Therefore, it is of great clinical significance to explore new molecular markers to effectively predict the recurrence and metastasis of liver cancer after the operation. In this study, we find that MBD3 is upregulated in HCC and elucidate its underlying mechanisms. We observed stronger MBD3-positive staining in HCC patients with a higher AFP level, no tumour capsules and poor cell differentiation. We also found that MBD3 expression was significantly correlated with advanced cancer biology, indicated by early vascular invasion and late clinical-stage TNM. Furthermore, the MBD3 staining intensity was also inversely associated with OS and DFS. Collectively, we provide the first evidence that MBD3 can be used as a novel biomarker for HCC patient outcomes after surgery.

We demonstrated that MBD3 could promote the proliferation, migration and invasion of HCC cells in vitro and in vivo. Several studies have demonstrated that MBD3 is reduced in gastric, pancreatic, colon and lung cancers, suggesting that its downregulation may contribute to carcinogenesis [11, 18, 19]. Li R, et al. have reported that suppression of MBD3, together with OSKM (Oct4, Sox2, Klf4 and c-Myc) transduction, induces the conversion of liver cancer cells into stem-like cells [20]. In this study, we reveal a critical role for MBD3 in the progression and metastasis of HCC.

The different roles of MBD3 in the development of different types of tumours may depend on the specific tumour type, tumour microenvironment and what molecule it interacts with. In this study, we identified TFPI2 as a downstream target gene of MBD3 in HCC using RNA-seq analysis. TFPI2 is a Kunitz-type serine proteinase inhibitor that has been previously identified as a tumour suppressor involved in numerous cancers [21–23]. It can prevent the proliferation, invasion and metastasis of malignant tumour cells by inhibiting the activity of matrix metalloproteinases (MMPs) [24, 25]. It is well known that the inactivation of tumour suppressor genes is a crucial event in the formation and progression of tumours [26]. Researchers have observed that a loss of TFPI2 expression in many malignancies can antagonise the development and progression of cancers.

More importantly, we also observed an inverse correlation between MBD3 expression and TFPI2 expression in human HCC consecutive specimens. Some studies have reported that TFPI2 may inhibit tumour metastasis by inhibiting tumour angiogenesis [16], but there is no convincing clinical evidence to support it. As a vascular endothelial marker, CD34 is often used to mark tumour angiogenesis [27]. Fortunately, we found that the expression of TFPI2 was negatively correlated with that of CD34 in serial sections of HCC. Based on these important findings, we conclude that MBD3 can inhibit the expression of TFPI2, indirectly relieve the inhibitory effect of TFPI2 on tumour angiogenesis, and then promote tumour metastasis.

Additionally, we demonstrated that MBD3 promotes HCC progression by inhibiting the tumour suppressor TFPI2 in vitro and in vivo. However, further research is still needed to explore how MBD3 regulates TFPI2 gene transcription and inhibits its protein expression. MBD3 has been reported as a core subunit of the NuRD co-repressor complex, which exerts its transcriptional repression function by recruiting other components such as HDAC1 [28, 29]. In our study, we further confirmed that MBD3 can recruit other subunits CHD4 and HDAC1 to form NuRD complex, and combine with the *TFPI2* promoter, then deacetylate TFPI2 promoter through HDAC1, which leads to gene silencing. MBD3 plays a tumour-promoting role in HCC through epigenetic regulation of TFPI2 transcription. Next, we also demonstrated that TFPI2 can inhibit the MMP1 and MMP10 in HCC, as well as the activation of the PI3K/AKT pathway. The main function of MMPs is to degrade extracellular material or matrix, thus promoting tumour cells proliferation and migration [30]. MMP1 and MMP10 mainly affect the migration, invasion and angiogenesis of tumour cells [31, 32]. Hyperactivation of PI3K/AKT signaling pathway is one of the most ordinary events in human cancers, which is mainly responsible for tumour cell proliferation [33]. Therefore, when TFPI2 was inhibited by MBD3, all the above proteins would be reactivated to promote the progress, angiogenesis and metastasis of HCC.

As a tumour suppressor, TFPI2 has been reported to be silenced through epigenetic modification and thereby contribute to tumour growth and metastasis [34–39]. Methylated TFPI2 has been considered a potential tumour marker for the early prediction of tumorigenesis, including HCC, colorectal cancer and oesophagal cancer [40–43]. However, besides DNA methylation, other epigenetic modifications affecting *TFPI2* gene transcription and protein expression, as well as its upstream regulatory molecules, have not been reported. Here, we found that TFPI2 can be regulated and deacetylated by the upstream MBD3/NuRD complex. MBD3, together with other subunits of the NuRD complex, bind to *TFPI2* and further deacetylate it through HDAC1, inhibiting the gene transcription of *TFPI2*. In fact, it has been reported that deacetylated histones tend to accumulate on hypermethylated DNA, while MBD3 is a methylated DNA binding protein and a component of NuRD complex, which cause heterochromatin remodelling and transcriptional inhibition through the activity of histone deacetylase complexes (HDACs) [44]. Some studies have demonstrated that DNA methylation and histone deacetylation act largely independently to suppress transcription factor binding and gene expression, and synergistically on some genes [45].

In summary, this study provided critical insight into the role of the MBD3 protein in the progression and metastasis of HCC. We identified that MBD3 plays a key role in promoting the proliferation, angiogenesis and invasion of HCC by inhibiting tumour suppressor TFPI2. We further demonstrated that MBD3 can deacetylate *TFPI2* promoter through HDAC1 in NuRD complex, leading to gene inhibition. These findings not only highlight MBD3 as a promising marker for HCC diagnosis and prognosis but also reveal a novel therapeutic target for clinical HCC treatment.

## Supplementary information

Supplementary Materials and Methods

Supplementary Figure S1-5

Supplementary Table S1

Supplementary Table S2

Supplementary Table S3

Supplementary Table S4

Supplementary Table S5

Supplementary Table S6

## Data Availability

All the data that support the findings of this study are available from the corresponding author upon reasonable request.
