# Supplementary material for: MBD3 promotes hepatocellular carcinoma progression and metastasis through negative regulation of tumour suppressor TFPI2
Source: Br J Cancer. 2022 Apr 30;127(4):612–23. doi: 10.1038/s41416-022-01831-5 (PMC9381593; doi:10.1038/s41416-022-01831-5)
Supplement: Supplementary file 1 — Supplementary Materials and Methods [file 41416_2022_1831_MOESM1_ESM.pdf]

Supplementary materials & methods

The source and calculation of TCGA data

We obtained 369 cases HCC with MBD3 expression, of which 160 had adjacent normal liver tissues from the UCSC Xena database (https://xenabrowser.net/hub/). The data was Batch effects normalized mRNA data by automatic analysis of the website, and the expression difference between HCC and normal liver tissues was statistically analyzed by the Mann-Whitney U test.

Plasmids and cell infection

The plasmids for control and overexpression of MBD3 are pCDH-MCS-T2A-copGFP-MSCV and pCDH-MCS-T2A-copGFP-MSCV-Flag-MBD3. The plasmids for control and knockdown of MBD3 are pLKO.1-puro-shNT and pLKO.1-puro-shMBD3-1#, pLKO.1-puro-shMBD3-2#. The plasmids for control and overexpression of sh-RNA resistant MBD3 are pCDH-MCS-T2A-puro-MSCV and pCDH-MCS-T2A-puro-MSCV-Flag-MBD3 (this plasmid was mutated with primer and the overexpression plasmid resistant to shRNA knockdown was obtained). The plasmids for control and knockdown of TFPI2 are pCDH-CMV-Neo-shNT, pCDH-CMV-Neo-shTFPI2 1# and pCDH-CMV-Neo-shTFPI2 2#. All the plasmids were stably infected into hepatoma cells by lentivirus.

Cell proliferation – Plate Colony-forming assay

Cells were digested and inoculated into a 6-well culture plate (2000 cells per well, evenly dispersed) and cultured at 37 °C and 5% CO_2. After 10 days of culture, when

the cell clones were visible to the naked eye (generally > 50 cells aggregated) the culture were terminated; the culture supernatant was discarded and carefully washed twice with PBS. They were then fixed in methanol for 15 min, washed twice with PBS, dyed with 0.4% crystal violet for 20 min, slowly washed again with PBS, and dried at room temperature. The culture plate was inverted and photographed, and clones were counted using Image-Pro Plus 6.0 software to calculate the cloning formation rate.

Cell proliferation – Soft agar colony-forming assay

2x medium was combined with 1.0% agar and 0.6% agar separately. Before the agar solution introduction high pressure should be applied to improve miscibility. Once the agar solutions are cool (37-40 °C), 5 mL 1% agar was mixed it with 5 mL of 2x medium to make 10 mL of 0.5% agar for the underlying gel. 1.5 mL of 0.5% agar was then added to each well in a 6-well plate and left coagulation. Cells were diluted to 15,000 cells/300 μL complete medium for reserve. Then, 5 mL 0.6% agar was mixed with 5 mL 2x medium to make 0.3% agar. 4.5 mL 0.3% agar was then slowly added into the above 300 μL diluted cells. 1.5 mL of the mixture was then added to each well of the previously paved 6-well plate (5000 cells per well). After culture at 37 °C and 5% CO_2 for 2 weeks, cell clone spheres were imaged and counted under microscope.

Cell migration – Transwell

Cells were digested and cultured in each chamber without laying Matrigel gel, with 4×10^4 cells in 100 μL serum-free medium and 500 μL complete medium in the lower holes. After cultured in 5% CO_2 at 37 °C for 24 h, the samples were collected and

the inner cells were removed with cotton swabs; then, the outer cells penetrating the chamber were immersed in 75% ethanol for 15 min, followed by 0.4% crystal violet solution for 15 min, and finally immersed in distilled water 2-3 min for 3 times. After drying, the bottom cells were scanned, photographed, and counted.

Cell migration − Wound healing assay

Cells were digested and inoculated in 6-well plates with 5×10^5 cells/well, cultured with complete medium, and grown overnight. The next day, cells were scratched with 10μl tips. Cells were then washed 3 times with PBS to remove the scratched cells, serum-free medium was then added in replacement. The wounded cells were then cultured in 5% CO_2 at 37 °C, with measurements and photographs of the wound healing taken at 0 h, 24 h and 48 h.

Immunofluorescence

The paraffin sections of nude mice subcutaneous tumors and clinical HCC patients were sent to the Panovue Biotechnology (Beijing) Co., Ltd for immunofluorescence staining experiments and data analysis. The antibodies used are as follows: MBD3 (14258-1-AP), TFPI2 (ab186747), MMP1 (ab137332), MMP10 (ab199688), CD34 (MA1-10202), CHD4 (12011S), HDAC1 (34589S). The results were expressed by H-score. H-score = [(cell positive rate with intensity of 1+) x 1 + (cell positive rate with intensity of 2+) x 2 + (cell positive rate with intensity of 3+) x 3] x 100.

Luciferase activity

Huh7-shNT cells and -shMBD3 cells were transiently co-transfected with 100 ng TFPI2 promoter-driven luciferase (TFPI2 Pro-Luc（-1000-0））and 10 ng pRL-TK renilla luciferase plasmid (internal control, Promega). 24 hours later, luciferase activities were measured with the Dual-Luciferase Reporter Assay system (E1910, Promega) according to the manufacturer’s instructions.

Whole genome methylation sequencing (WGBS)

WGBS and Data Analysis: three samples of Huh7-shNT cells and three samples of Huh7-shMBD3 cells were cultured and harvested for WGBS experiments. WGBS was conducted by the Annoroad Gene Technology Co. (Beijing, China). Briefly, 1μg DNA per sample was used to perform library construction. End-repair, A-tailing, ligation of adaptors and subsequent bisulfite conversion of the sequencing libraries was performed using DNA bisulfite convert kit (TIANGEN, Beijing) and then amplifying by PCR. The libraries were subjected to paired-end 150 bp sequencing using Nova-seq platform (Illumina). CpG methylation sites were identified with bismark (v0.21.0) against human genome hg38 and following analysis and visualization was performed with R.

Supplementary figure legend

Supplementary Fig. S1

MBD3 promotes HCC cell proliferation and migration *in vitro*.

(a and b) The effect of MBD3 on the growth of HCC cells was detected by the plate cloning experiments (a) and soft agar cloning experiments, Scale bar: 500μm (b). **(c and d)** The effect of MBD3 on the migration of hepatoma cells was detected by the transwell assay, scale bar: 100 μm (c), and wound healing assay, scale bar: 200μm (d). Data are represented as mean ± SD, assayed by unpaired Student's *t* test or Welch's *t* test. ***$P < 0.001$.

Supplementary Fig. S2

MBD3 promotes HCC cell proliferation and migration by inhibiting TFPI2.

(a) Hep3B cells were stably transfected with Control, MBD3 or Control + TFPI2, MBD3 + TFPI2, and the indicated protein expression levels were detected by western blotting. **(b and c)** The proliferation and invasion ability of the indicated cells were tested by MTS assay (b) and transwell assay (c, scale bar: 100μm). **(d and e)** Representative image of the subcutaneous tumorigenesis in nude mice. Tumor weight (d) and tumor growth curve (e) of the indicated groups (n=5/group) were analyzed. (b and e) Mean ± SD, two-way ANOVA; (c) Mean ± SD, (d) mean ± SEM, unpaired Student's t test or Welch's t test. *$P < 0.05$, **$P < 0.01$, ***$P < 0.001$.

Supplementary Fig. S3

MBD3 promotes HCC cell proliferation by activating PI3K-Akt signaling.

Hep3B-MBD3 cells were incubated with and without PI3K inhibitor LY294002, and the proliferation of the indicated cells were tested by MTS assay. Data are represented as Mean ± SD, two-way ANOVA. *P < 0.05, **P < 0.01, ***P < 0.001.

Supplementary Fig. S4

MBD3/NuRD complex binds to the promoter of TFPI2.

(a) The endogenous association of CHD4 and HDAC1 is detected in Huh7 cells. Immunoprecipitation (IP) was performed using anti-HDAC1 antibody and the immunoprecipitates (IPs) were probed with indicated antibodies. **(b)** HDAC1 is knocked down in Huh7 cells to destroy the HDAC1/CHD4 complex and the expression level of MBD3 is detected by Western blotting. **(c)** The TFPI2 promoter-reporter construct (TFPI2-Luc) is shown on top. Transcriptional activation of TFPI2 was measured using a TFPI2 promoter luciferase reporter assay (bottom). Luciferase activity was measured 60 h after transfection, and activity was normalized to the level of shNT expression. Data are represented as Mean ± SD, unpaired Student's t test. *P < 0.05, **P < 0.01, ***P < 0.001.

Supplementary Fig. S5

MBD3 has no effect on the methylation of TFPI2.

(a) The DNA methylation of Huh7 cells in shNT and shMBD3 groups was detected by the Whole genome methylation sequencing (WGBS). **(b)** Effect of MBD3 on methylation ratio of TFPI2 promoter region. The methylation ratio at the TFPI2 promoter (-1000 to +1000 bp) is analyzed by Mann Whitney test, *P* value = 0.2458.
