# Supplementary material for: MBD3 promotes hepatocellular carcinoma progression and metastasis through negative regulation of tumour suppressor TFPI2
Source: Br J Cancer. 2022 Apr 30;127(4):612–23. doi: 10.1038/s41416-022-01831-5 (PMC9381593; doi:10.1038/s41416-022-01831-5)
Supplement: Supplementary file 2 — Supplementary Figure S1-5 [file 41416_2022_1831_MOESM2_ESM.pdf]

Supplementary Fig. S1

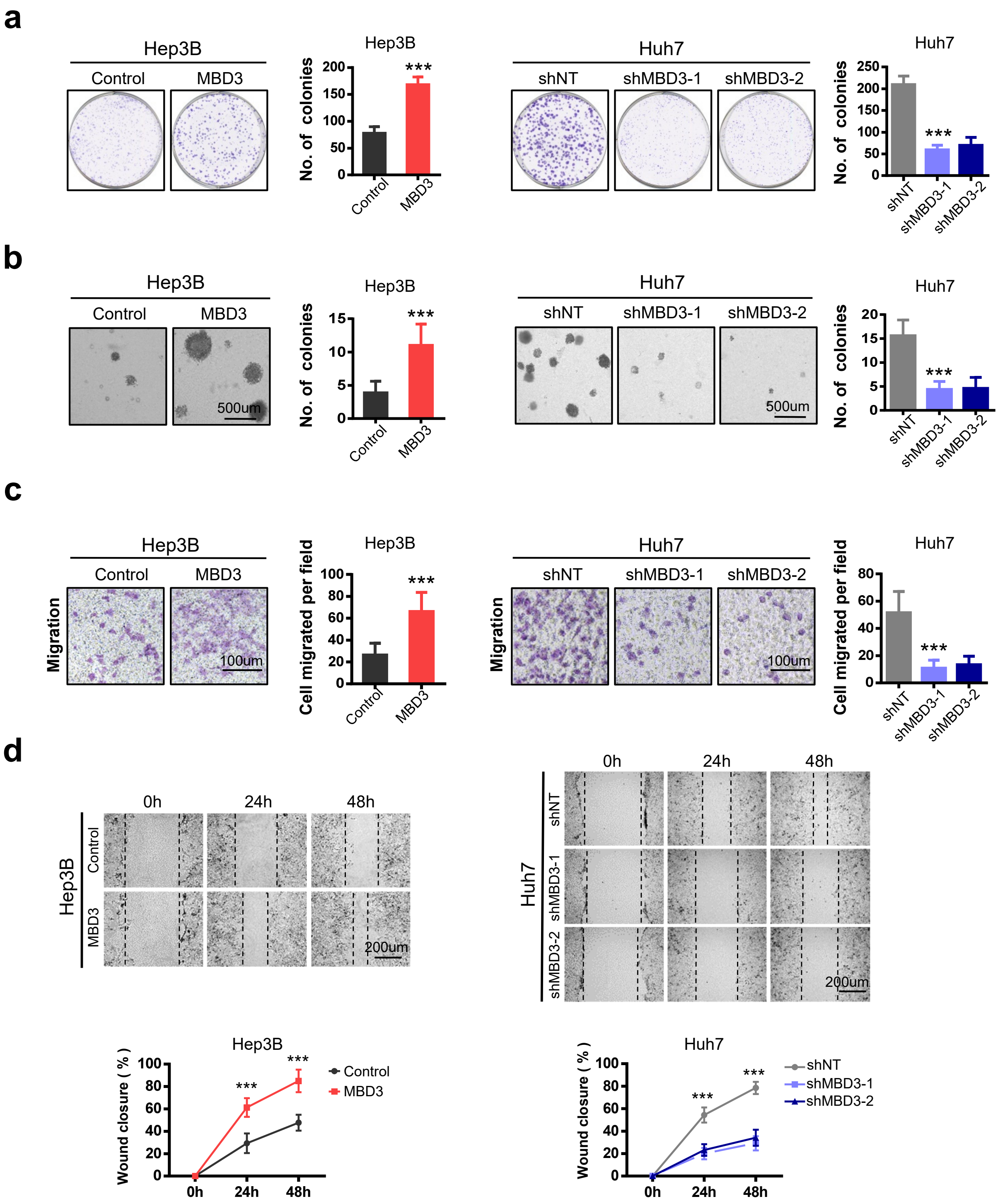

Supplementary Fig. S1 MBD3 promotes HCC cell proliferation and migration *in vitro*.

(a and b) The effect of MBD3 on the growth of HCC cells was detected by the plate cloning experiments (a) and soft agar cloning experiments (b, scale bar: 500μm). **(c and d)** The effect of MBD3 on the migration of hepatoma cells was detected by the transwell assay (c, scale bar: 100μm), and wound healing assay (d, scale bar: 200μm). Data are represented as mean ± SD, assayed by unpaired Student's *t* test or Welch's *t* test. ***$P < 0.001$.

Supplementary Fig. S2

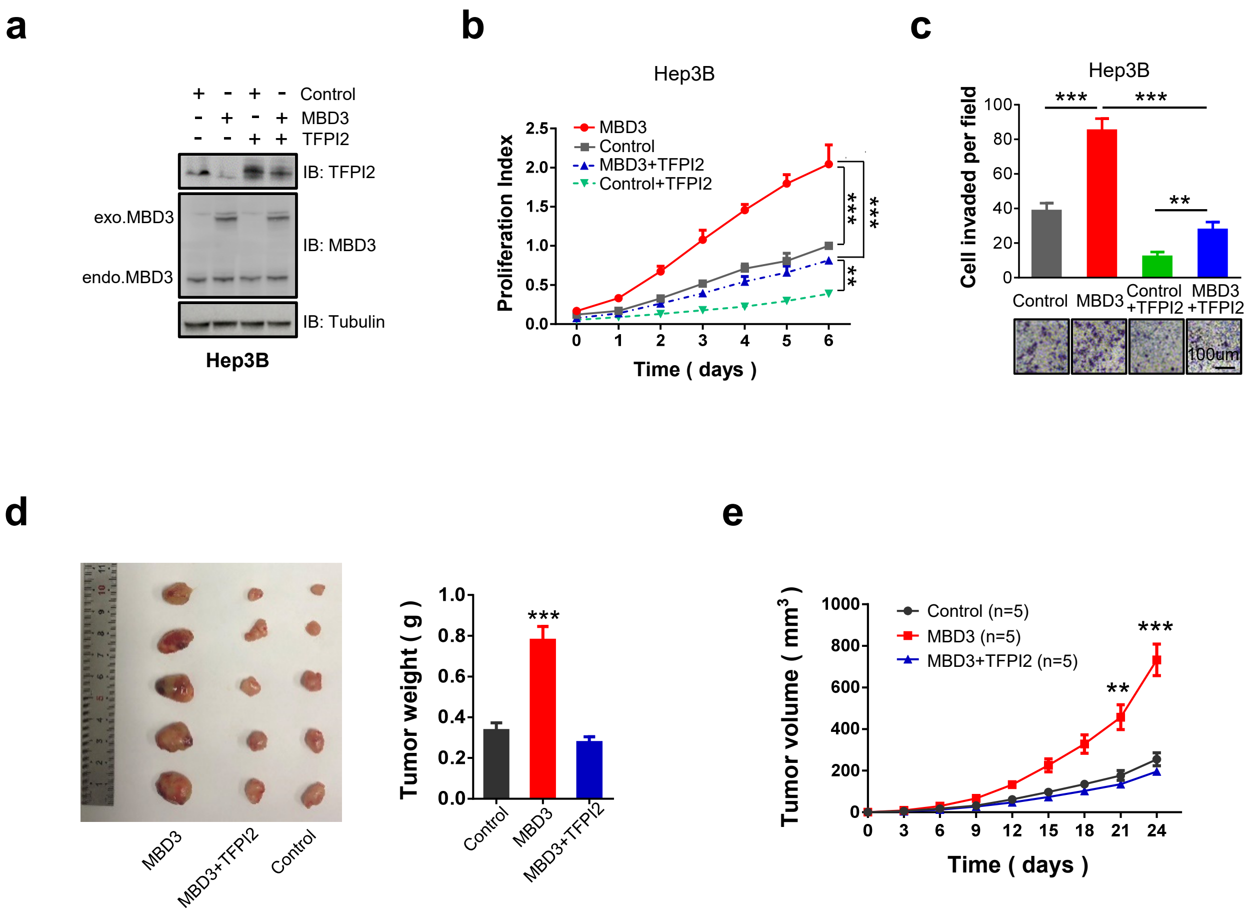

Supplementary Fig. S2 MBD3 promotes HCC cell proliferation and migration by inhibiting TFPI2.

(a) Hep3B cells were stably transfected with Control, MBD3 or Control + TFPI2, MBD3 + TFPI2, and the indicated protein expression levels were detected by western blotting. **(b and c)** The proliferation and invasion ability of the indicated cells were tested by MTS assay (b) and transwell assay (c, scale bar: 100μm). (**d and e)** Representative image of the subcutaneous tumorigenesis in nude mice. Tumor weight (d) and tumor growth curve (e) of the indicated groups (n=5/group) were analyzed. (b and e) Mean ± SD, two-way ANOVA; (c) Mean ± SD, (d) mean ± SEM, unpaired Student's t test or Welch's t test. *P$<$0.05, **P$<$0.01, ***P$<$0.001.

Supplementary Fig. S3

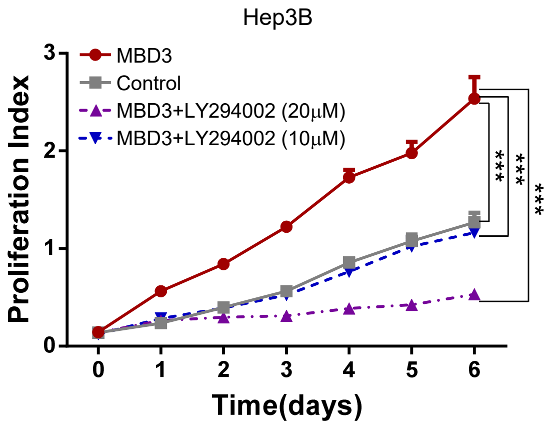

Supplementary Fig. S3 MBD3 promotes HCC cell proliferation by activating PI3K-Akt signaling.

Hep3B-MBD3 cells were incubated with and without PI3K inhibitor LY294002, and the proliferation of the indicated cells were tested by MTS assay. Data are represented as Mean ± SD, two-way ANOVA. *P＜0.05, **P＜0.01, ***P＜0.001.

Supplementary Fig. S4

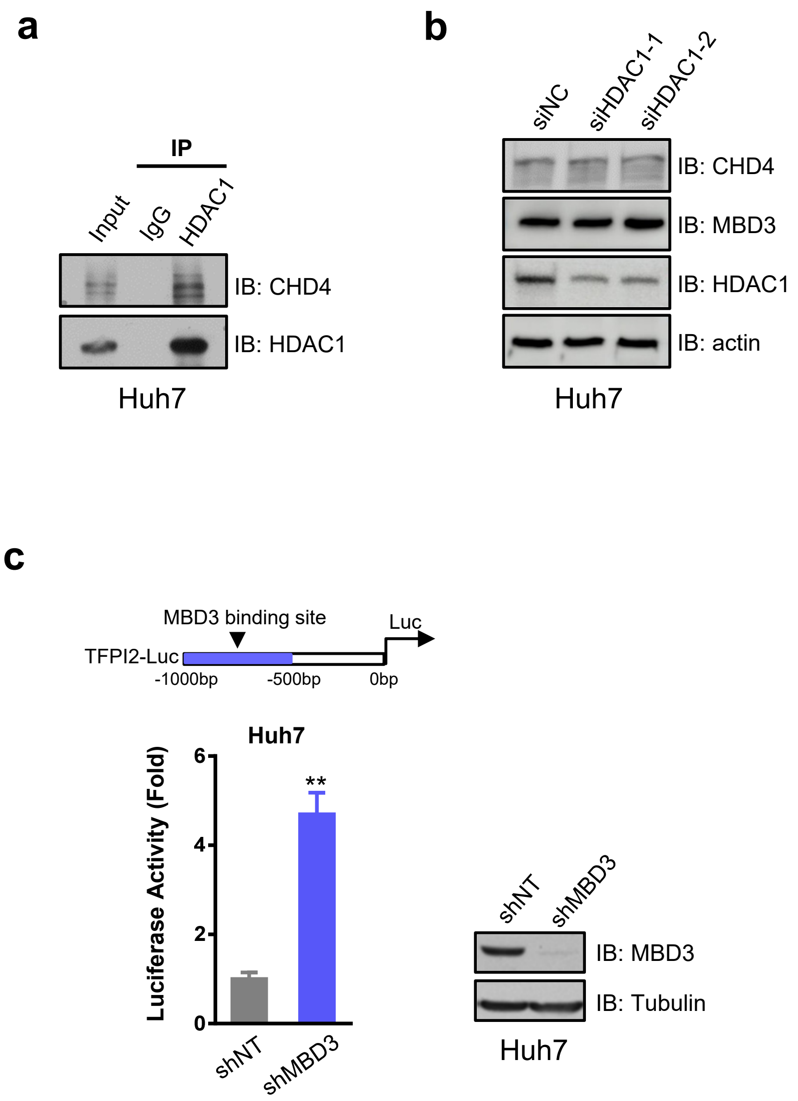

Supplementary Fig. S4 MBD3/NuRD complex binds to the promoter of TFPI2.

(a) The endogenous association of CHD4 and HDAC1 is detected in Huh7 cells. Immunoprecipitation (IP) was performed using anti-HDAC1 antibody and the immunoprecipitates (IPs) were probed with indicated antibodies. **(b)** HDAC1 is knocked down in Huh7 cells to destroy the HDAC1/CHD4 complex and the expression level of MBD3 is detected by Western blotting. **(c)** The TFPI2 promoter-reporter construct (TFPI2-Luc) is shown on top. Transcriptional activation of TFPI2 was measured using a TFPI2 promoter luciferase reporter assay (bottom). Luciferase activity was measured 60 h after transfection, and activity was normalized to the level of shNT expression. Data are represented as Mean ± SD, unpaired Student's t test. *$P < 0.05$, **$P < 0.01$, ***$P < 0.001$.

Supplementary Fig. S5

a

sample	Total read pair (million)	Number of methylated CpG (million)	Percentage of analyzed CpG
shNT-1	349.4	283.1	68.6%
shNT-2	381.7	311.8	69.3%
shNT-3	434.9	347.8	68.7%
shMBD3-1	388.5	304.2	69.1%
shMBD3-2	374.8	309.7	69.3%
shMBD3-3	250.5	203.6	68.9%

b

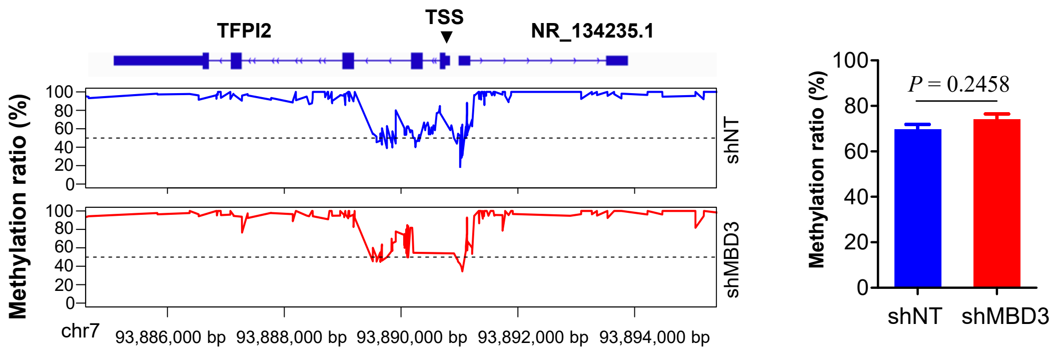

Supplementary Fig. S5 MBD3 has no effect on the methylation of TFPI2.

(a) The DNA methylation of Huh7 cells in shNT and shMBD3 groups was detected by the Whole genome methylation sequencing (WGBS). **(b)** Effect of MBD3 on methylation ratio of TFPI2 promoter region. The methylation ratio at the TFPI2 promoter (-1000 to +1000 bp) is analyzed by Mann Whitney test, *P* value = 0.2458.
