# Supplementary material for: MBD3 promotes hepatocellular carcinoma progression and metastasis through negative regulation of tumour suppressor TFPI2
Source: Br J Cancer. 2022 Apr 30;127(4):612–23. doi: 10.1038/s41416-022-01831-5 (PMC9381593; doi:10.1038/s41416-022-01831-5)
Supplement: Supplementary file 3 — Supplementary Table S1 [file 41416_2022_1831_MOESM3_ESM.docx]

**Supplementary Table S1. All the antibodies used in this study.**

| **Experiment** | **Antibody** | **Manufacturer** | **Catalog Number** | **Concentration** |
| --- | --- | --- | --- | --- |
| **IHC** | MBD3 | Proteintech | 14258-1-AP | 1:200 |
|  | TFPI2 | Abcam | ab186747 | 1:200 |
|  | CD34 | Thermo Fisher Scientific | MA1-10202 | 1:500 |
| **Western blot** | MBD3 | Proteintech | 14258-1-AP | 1:1000 |
|  | TFPI2 | Abcam | ab186747 | 1:1000 |
|  | MMP1 | Abcam | ab137332 | 1:1000 |
|  | MMP10 | Abcam | ab199688 | 1:1000 |
|  | PI3K | Abcam | ab86714 | 1:1000 |
|  | p-PI3K | Abcam | ab182651 | 1:500 |
|  | AKT | Abcam | ab179463 | 1:1000 |
|  | p-AKT | CST | 4060s | 1:2000 |
|  | Flag | Sigma | F3165 | 1:5000 |
|  | Tubulin | Sigma | T5168 | 1:5000 |
| **ChIP** | MBD3 | Abcam | ab91458 | 1:50 |
|  | HDAC1 | CST | 34589s | 1:50 |
|  | CHD4 | CST | 12011s | 1:50 |
|  | H3K27me3 | CST | 9733s | 1:50 |
|  | H3K27Ac | CST | 8173s | 1:50 |
| **Co-IP** | MBD3 | Abcam | ab91458 | 2-10ug/mg |
| **IF** | MBD3 | Proteintech | 14258-1-AP | 1:50 |
|  | CHD4 | CST | 12011s | 1:400 |
|  | HDAC1 | CST | 34589s | 1:100 |
|  | TFPI2 | Santa Cruz | sc-48380 | 1:200 |
|  | MMP1 | Abcam | ab137332 | 1:200 |
|  | MMP10 | Abcam | ab261733 | 1:50 |
|  | CD34 | Thermo Fisher Scientific | MA1-10202 | 1:400 |
