# Supplementary material for: MBD3 promotes hepatocellular carcinoma progression and metastasis through negative regulation of tumour suppressor TFPI2
Source: Br J Cancer. 2022 Apr 30;127(4):612–23. doi: 10.1038/s41416-022-01831-5 (PMC9381593; doi:10.1038/s41416-022-01831-5)
Supplement: Supplementary file 4 — Supplementary Table S2 [file 41416_2022_1831_MOESM4_ESM.docx]

**Supplementary Table S2. All the primers used in the plasmids.**

| Nhe1-G-F-MBD3 | CGGCTAGCCACCATGGTGAGCAAGGGCGAGGAG |
| --- | --- |
| Not1-Nhe1-G-F-MBD3 | TTGCGGCCGCCTAGACGTGCTCCATCTC |
| sh-MBD3-1-F | CCGGCAAGATGCTGATGAGCAAGATCTCGAGATCTTGCTCATCAGCATCTTGTTTTTG |
| sh-MBD3-1-R | AATTCAAAAACAAGATGCTGATGAGCAAGATCTCGAGATCTTGCTCATCAGCATCTTG |
| sh-MBD3-2-F | CCGGCCTGTGCAAAGCCTTCATGGTCTCGAGACCATGAAGGCTTTGCACAGGTTTTTG |
| sh-MBD3-2-R | AATTCAAAAACCTGTGCAAAGCCTTCATGGTCTCGAGACCATGAAGGCTTTGCACAGG |
| MBD3-resis-1-F | GGAGATGGAGCACGTCTACAAGGCGGCCGCT |
| MBD3-resis-1-R | TGTAGACGTGCTCCATCTCCGGGTCCGGGTC |
| MBD3-resis-2-F | CGGAGATGGAGCACGTCGCGGCCGCTGAGGGC |
| MBD3-resis-2-R | CGCGACGTGCTCCATCTCCGGGTCCGGGTCGG |
| luciferase-F | GAAGACGCCAAAAACATAAAG |
| luciferase-R | CAATTTGGACTTTCCGCCCTT |
| shTFPI2-1-F | CCGGGGAACCGGATTGAGAACAGTTCAAGAGACTGTTCTCAATCCGGTTCCTTTTTTG |
| shTFPI2-1-R | AATTCAAAAAGGAACCGGATTGAGAACAGTTCAAGAGACTGTTCTCAATCCGGTTCCT |
| shTFPI2-2-F | CCGGCCGGATTGAGAACAGGTTTCTCAAGAGAAAACCTGTTCTCAATCCGGTTTTTTG |
| shTFPI2-2-R | AATTCAAAAACCGGATTGAGAACAGGTTTCTCAAGAGAAAACCTGTTCTCAATCCGGT |
| TFPI2-F | CTAGCTAGCGCCACCATGGACCCCGCTCGCCCCCTGG |
| TFPI2-R | AATGCGGCCGCTTACTTATCGTCGTCATCCTTGTAATCAAATTGCTTCTTCCGAATTTTC |
