# Supplementary material for: MBD3 promotes hepatocellular carcinoma progression and metastasis through negative regulation of tumour suppressor TFPI2
Source: Br J Cancer. 2022 Apr 30;127(4):612–23. doi: 10.1038/s41416-022-01831-5 (PMC9381593; doi:10.1038/s41416-022-01831-5)
Supplement: Supplementary file 5 — Supplementary Table S3 [file 41416_2022_1831_MOESM5_ESM.docx]

**Supplementary Table S3. All the primers used in the ChIP.**

| **Name** | **Sequence** (5'→3') | **Length** |
| --- | --- | --- |
| 1#-TFPI2-F | 5'-TTTTCCCACCTCGGCATTC-3' | 19 |
| 1#-TFPI2-R | 5'-GATTTCTGTGATGTAAATGTGCTCACT-3' | 27 |
| 2#-TFPI2-F | 5'-AGGAAATGTGTGGGAATACACTGA-3' | 24 |
| 2#-TFPI2-R | 5'-AAGAACCATAGCAGCCCAGAGA-3' | 22 |
| 3#-TFPI2-F | 5'-TTGCCCCTGGCCTAAGG-3' | 17 |
| 3#-TFPI2-R | 5'-GTTAGAACATTTCCATCACTCCAGAA-3' | 26 |
| 4#-TFPI2-F | 5'-CTCCGCTCTGACCCAAGAAC-3' | 20 |
| 4#-TFPI2-R | 5'-TCCACCTCTTGAAGGCATGAA-3' | 21 |
| 5#-TFPI2-F | 5'-GTCTGGACTACAGGAGAAAGTTTGG-3' | 25 |
| 5#-TFPI2-R | 5'-CAAACTGTGTAAGAGGGAGAGGAATT-3' | 26 |
| 6#-TFPI2-F | 5'-GAGGGCAACGCCAACAATT-3' | 19 |
| 6#-TFPI2-R | 5'-CGCGCAGGGCACTTACTTT-3' | 19 |
