# Supplementary material for: MBD3 promotes hepatocellular carcinoma progression and metastasis through negative regulation of tumour suppressor TFPI2
Source: Br J Cancer. 2022 Apr 30;127(4):612–23. doi: 10.1038/s41416-022-01831-5 (PMC9381593; doi:10.1038/s41416-022-01831-5)
Supplement: Supplementary file 6 — Supplementary Table S4 [file 41416_2022_1831_MOESM6_ESM.docx]

**Supplementary Table S4.** **Clinicopathological correlation of MBD3 expression in human HCCs.**

| **Clinicopathological features** | Number | MBD3 high expression | MBD3 low expression | $\chi$^2^ | *P* value |
| --- | --- | --- | --- | --- | --- |
| **N（%）** |  | 116(51.1%) | 111(48.9%) |  |  |
| ***Gender*** |  |  |  |  |  |
| Male | 196 | 101 | 95 | 0.106 | 0.745 |
| Female | 31 | 15 | 16 |  |  |
| ***Age(years)*** |  |  |  | 1.488 | 0.223 |
| ≤50 | 136 | 74 | 62 |  |  |
| ＞50 | 91 | 42 | 49 |  |  |
| **Alcohol** |  |  |  | 1.274 | 0.259 |
| Yes | 48 | 28 | 20 |  |  |
| No | 179 | 88 | 91 |  |  |
| ***HBsAg*** |  |  |  | 1.274 | 0.259 |
| Positive | 211 | 110 | 101 |  |  |
| Negative | 16 | 6 | 10 |  |  |
| ***Liver cirrhosis*** |  |  |  | 0.252 | 0.616 |
| Present | 187 | 97 | 90 |  |  |
| Absent | 40 | 19 | 21 |  |  |
| ***AFP level(μg/L)*** |  |  |  | 12.271 | 0.000 |
| ≤400 | 102 | 39 | 63 |  |  |
| ＞400 | 125 | 77 | 48 |  |  |
| ***Vascular invasion*** |  |  |  | 6.127 | 0.013 |
| Present | 46 | 31 | 15 |  |  |
| Absent | 181 | 85 | 96 |  |  |
| ***Lymphatic metastasis*** |  |  |  | 0.004 | 0.949 |
| Present | 8 | 4 | 4 |  |  |
| Absent | 219 | 112 | 107 |  |  |
| ***Tumor diameter*** |  |  |  | 0.119 | 0.731 |
| ≤5cm | 72 | 38 | 34 |  |  |
| ＞5cm | 155 | 78 | 77 |  |  |
| ***Tumor number*** |  |  |  | 0.647 | 0.421 |
| Single | 160 | 79 | 81 |  |  |
| Multiple | 67 | 37 | 30 |  |  |
| ***Tumor capsule*** |  |  |  | 6.111 | 0.013 |
| Present | 154 | 70 | 84 |  |  |
| Absent | 73 | 46 | 27 |  |  |
| ***Edmondson grade*** |  |  |  | 8.050 | 0.005 |
| I-II | 193 | 91 | 102 |  |  |
| III-IV | 34 | 25 | 9 |  |  |
| ***TNM stage*** |  |  |  | 10.303 | 0.001 |
| I-II | 131 | 55 | 76 |  |  |
| III-IV | 96 | 61 | 35 |  |  |

HCC, hepatocellular carcinoma; AFP, alpha-fetoprotein; HBsAg, hepatitis B virus surface antigen; TNM, tumor-node-metastasis; N, number.
