# Supplementary material for: MBD3 promotes hepatocellular carcinoma progression and metastasis through negative regulation of tumour suppressor TFPI2
Source: Br J Cancer. 2022 Apr 30;127(4):612–23. doi: 10.1038/s41416-022-01831-5 (PMC9381593; doi:10.1038/s41416-022-01831-5)
Supplement: Supplementary file 7 — Supplementary Table S5 [file 41416_2022_1831_MOESM7_ESM.docx]

**Supplementary Table S5. Univariate and multivariate analysis of factors associated with OS.**

|  | Univariate analysis | 95% CI |  | Multivariate analysis | 95% CI |  |
| --- | --- | --- | --- | --- | --- | --- |
| Parameters | Hazard ratio | Lower - Upper | *P* value | Hazard ratio | Lower - Upper | *P* value |
| MBD3 (high/low) | 2.298 | 1.652-3.197 | <0.001 | 1.883 | 1.337-2.650 | <0.001 |
| Gender (female/male) | 0.732 | 0.442-1.212 | 0.225 |  |  |  |
| Age (>50/≤50 years) | 0.780 | 0.562-1.083 | 0.138 |  |  |  |
| Alcohol (yes/no) | 1.172 | 0.796-1.724 | 0.422 |  |  |  |
| HBsAg (positive/negative) | 1.011 | 0.532-1.920 | 0.974 |  |  |  |
| Liver cirrhosis (present/absent) | 1.274 | 0.824-1.972 | 0.276 |  |  |  |
| AFP level (>400/≤400μg/L) | 1.613 | 1.162-2.237 | 0.004 |  |  |  |
| Vascular invasion (present/absent) | 2.900 | 1.998-4.208 | <0.001 |  |  |  |
| lymphatic metastasis (present/absent) | 4.751 | 2.297-9.830 | <0.001 | 3.724 | 1.746-7.945 | 0.001 |
| Tumor diameter (>5/≤5cm) | 1.999 | 1.377-2.901 | <0.001 |  |  |  |
| Tumor number (multiple/single) | 1.608 | 1.150-2.249 | 0.005 |  |  |  |
| Tumor capsule (present/absent) | 0.425 | 0.306-0.590 | <0.001 | 0.595 | 0.420-0.843 | 0.004 |
| Edmondson grade (III-V/ I-II) | 1.753 | 1.139-2.699 | 0.011 |  |  |  |
| TNM stage (III/I-II) | 3.332 | 2.399-4.628 | <0.001 | 2.421 | 1.698-3.454 | <0.001 |

HBsAg, hepatitis B virus surface antigen; AFP, alpha-fetoprotein; TNM, tumor-node-metastasis.
