# Supplementary material for: MBD3 promotes hepatocellular carcinoma progression and metastasis through negative regulation of tumour suppressor TFPI2
Source: Br J Cancer. 2022 Apr 30;127(4):612–23. doi: 10.1038/s41416-022-01831-5 (PMC9381593; doi:10.1038/s41416-022-01831-5)
Supplement: Supplementary file 8 — Supplementary Table S6 [file 41416_2022_1831_MOESM8_ESM.docx]

**Supplementary Table S6. Univariate and multivariate analysis of factors associated with DFS.**

|  | Univariate analysis | 95% CI |  | Multivariate analysis | 95% CI |  |
| --- | --- | --- | --- | --- | --- | --- |
| Parameters | Hazard ratio | Lower - Upper | *P* value | Hazard ratio | Lower - Upper | *P* value |
| MBD3 (high/low) | 1.970 | 1.450-2.676 | <0.001 | 1.645 | 1.201-2.254 | 0.002 |
| Gender (female/male) | 0.753 | 0.477-1.189 | 0.223 |  |  |  |
| Age (>50/≤50 years) | 0.825 | 0.609-1.119 | 0.216 |  |  |  |
| Alcohol (yes/no) | 1.298 | 0.911-1.849 | 0.149 |  |  |  |
| HBsAg (positive/negative) | 0.929 | 0.516-1.670 | 0.805 |  |  |  |
| Liver cirrhosis (present/absent) | 1.352 | 0.891-2.052 | 0.157 |  |  |  |
| AFP level (>400/≤400μg/L) | 1.537 | 1.135-2.082 | 0.005 |  |  |  |
| Vascular invasion (present/absent) | 2.774 | 1.929-3.989 | <0.001 |  |  |  |
| lymphatic metastasis (present/absent) | 3.794 | 1.842-7.814 | <0.001 | 2.644 | 1.258-5.556 | 0.010 |
| Tumor diameter (>5/≤5cm) | 1.521 | 1.098-2.107 | 0.012 |  |  |  |
| Tumor number (multiple/single) | 1.624 | 1.181-2.234 | 0.003 |  |  |  |
| Tumor capsule (present/absent) | 0.468 | 0.342-0.641 | <0.001 | 0.637 | 0.458-0.886 | 0.007 |
| Edmondson grade (III-V/ I-II) | 1.521 | 1.013-2.284 | 0.043 |  |  |  |
| TNM stage (III/I-II) | 3.089 | 2.263-4.215 | <0.001 | 2.394 | 1.711-3.348 | <0.001 |

HBsAg, hepatitis B virus surface antigen; AFP, alpha-fetoprotein; TNM, tumor-node-metastasis.
